# Object Recognition-Based Grasping with a Soft Modular Gripper

**DOI:** 10.3390/biomimetics11050347

**Published:** 2026-05-15

**Authors:** Yu Zhang, Fengwen Zhang, Zhihui Guo, Lingkai Luan, Dongbao Sui, Tianshuo Wang, Jiangyu Zhou, Fuyue Zhang, Chen Chen, Dongjie Li, Bo You

**Affiliations:** 1 Heilongjiang Provincial Key Laboratory of Complex Intelligent System and Integration, Harbin University of Science and Technology, Harbin 150080, China; 2 Ji Hua Laboratory, Foshan 528200, China

**Keywords:** soft modular gripper, deformation modeling, object recognition, YOLOv8

## Abstract

Soft modular grippers play a significant role in multiple fields due to their excellent adaptability and flexibility. This paper proposes a modular soft modular gripper driven by pneumatically actuated multi-chambers. The designed soft modular gripper features three operational modes, with its modular fingers employing independently controlled dual chambers. The distal and proximal dual-chamber structure enhances the fingertip force of the modular fingers. Based on classical laminated plate theory and incorporating the large deformation characteristics of soft materials, a relationship between the bending centerline of the fingers and the driving pressure is established, providing a theoretical foundation for grasping tasks executed by the soft modular gripper. The Denavit-Hartenberg (D-H) parameter method is utilized to develop the coordinate system of the soft modular gripper, thereby defining its operational workspace. Visual sensing technology is introduced, incorporating improvements to the YOLOv8-based object recognition and localization framework, which enhances recognition accuracy for target objects and ensures grasping stability.

## 1. Introduction

With the continuous advancement of soft robotics technology, bionic soft modular grippers have gradually played significant roles in various fields including industry, healthcare, and agriculture. Compared to traditional rigid robotic hands, soft modular grippers exhibit greater flexibility and adaptability, enabling them to effortlessly grasp fragile objects [1,2]. Yan et al. designed a soft robotic gripper with modular anthropomorphic fingers, achieving precise object grasping [3]. Zhang et al. proposed a modular soft gripper combining conventional and chevron actuator functions, enabling multiple grasping modes [4]. Chu et al. introduced a novel soft robotic finger demonstrating high adaptability in grasping [5]. Huang et al. developed a morphologically configurable soft gripper capable of high-load grasping [6]. Cui et al. presented a pneumatic soft-rigid hybrid multi-fingered gripper that adjusts postures to grasp objects of various sizes and weights [7]. Fang et al. proposed a soft gripper with multiple grasping modes through object enveloping or adsorption [8]. Roh et al. designed a millimeter-scale soft gripper based on shape-memory polymers, achieving object grasping through variable stiffness [9]. Tawk et al. developed a modular soft gripper capable of performing diverse grasping and holding tasks [10]. Seibel et al. introduced a soft gripper composed of six fluidic soft bending actuators, suitable for planar objects or those with at least one flat surface [11]. Xu et al. proposed a pneumatic fingerless soft gripper that manipulates objects by altering internal pneumatic chamber pressure and structure [12]. Zhao P et al. presented a modular soft gripper with variable stiffness for grasping objects of different structures and materials [13]. Wu M et al. developed an octopus-sucker-inspired soft gripper capable of grasping out-of-reach, irregular, and scattered objects [14]. Jain S et al. designed a reconfigurable soft gripper enabling multiple grasping modes and workspace reconfiguration [15]. Faris et al. proposed a 3D-printed soft pneumatic finger that achieves object grasping through sensor-measured pressure changes [16].

The integration of target detection and object recognition technologies has enhanced the adaptive grasping capabilities and accuracy of soft modular grippers. Cheng et al. designed a modular reconfigurable soft gripper that automatically controls grasping postures and dimensions by recognizing fruit shapes and sizes [17]. Matos et al. proposed a modular mechanical gripper incorporating embedded cameras and computer vision for object recognition [18]. Zhang et al. developed a soft gripper with bionic optical tactile palms, achieving stable grasping and precise placement [19]. Almanzor et al. designed a soft modular gripper utilizing deep learning-based grasp classification for objects of various sizes and shapes [20]. Zhang et al. created a soft modular gripper with integrated perception functions, improving grasping stability and object classification accuracy [21]. Chen et al. introduced a soft modular gripper integrating vision and soft tactile sensors, achieving estimation of object size and stiffness [22].

Despite the significant progress of soft grippers, several key challenges remain unresolved. First, some existing structural designs only provide limited grasping modes and lack diverse actuation methods, which limits their adaptability to different objects. By analyzing the bionic principles of human fingers and the grasping process, the design of optimized finger structures to achieve superior grasping performance still requires further exploration and innovation. Second, current studies rarely establish a unified and accurate theoretical model of bending deformation, driving pressure, and workspace, making it difficult to accurately obtain the operating space of the soft hand. Finally, although object recognition algorithms have developed rapidly and provided more methods for object recognition and localization, utilizing existing image recognition algorithms and improving them to build a vision-based integrated operating platform still requires further research. A comparison with several related soft gripper studies is summarized in Table 1.

In this paper, a distal-proximal dual-chamber modular finger is proposed to enhance fingertip grasping force, and three control modes are achieved to flexibly adapt to the grasping of different objects. A theoretical model is established by combining deformation modeling based on classical laminate theory, constant-curvature deformation analysis, and workspace analysis using the D-H parameter method, revealing the nonlinear relationship between bending deformation and driving pressure, which provides a theoretical basis for grasping control. The YOLOv8 algorithm is improved by integrating multi-scale convolution modules and attention mechanisms to enhance recognition accuracy, and the recognition, classification, localization, and pose acquisition of target objects are completed to provide guidance for reliable grasping. A comprehensive grasping system platform is constructed with the integration of vision, soft grippers, and pneumatic control, integrating visual sensors, modular soft grippers, object detection and localization, driving units, mobile grasping methods, and a human–computer interaction interface.

## 2. Design of Soft Modular Gripper

**Figure 1:**
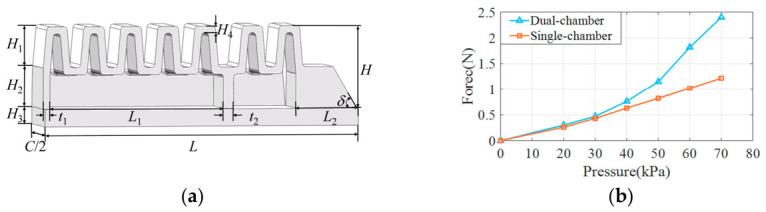

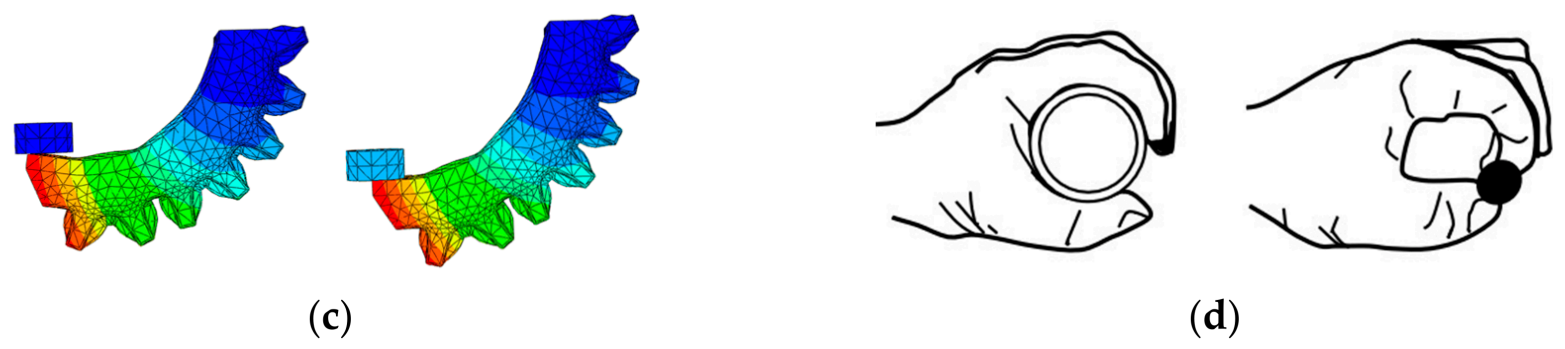
Design of soft finger structure: (**a**) Finger structure parameters. (**b**) End-effector force comparison curve. (**c**) Simulation of single-chamber actuation and dual-chamber actuation. (**d**) Grasping types illustration: grasping and pinching.

The soft modular gripper consists of three modular fingers, each sharing the same structural design. As shown in Figure 1a, each finger is designed as a pneumatic dual-chamber structure, divided into a distal and a proximal segment. Simulation is conducted using ABAQUS 2021. The geometric models of each component are established using SOLIDWORKS and imported into the ABAQUS finite element analysis software. In ABAQUS, the material parameters are defined firstly, where the hyperelastic Yeoh model is selected with coefficients of 0.038 and 0.013. Subsequently, corresponding section properties are created and assigned to each component, and the assembly and positioning of components are realized by constructing constraint relationships among points, lines and surfaces. A static general analysis step is adopted with geometric nonlinearity enabled, and the full Newton method is applied as the solution technique. The boundary condition is set to fix the upper end of the soft finger, and pressure loads are applied to the interior of the cavity. After setting the assembly instance as independent, mesh control attributes are assigned, and then mesh seeding and division are carried out. The element type selected is C3D10H with second-order geometry and hybrid formulation selected. Finally, an analysis job is created and the complete analysis is submitted using the standard implicit solver.

Under the same structural parameters, the fingertip force is enhanced by the dual-chamber design. The structural and simulation parameters of the soft robotic finger are shown in Table 2. The finger contains two pneumatic chambers, enabling two types of grasping motions similar to those of a human hand. As illustrated in Figure 1d, simultaneous actuation of both chambers results in a full grasp, while actuating only the distal chamber enables a pinch grasp.

As shown in Figure 1c, in the simulation environment, an internal pressure load is applied to the model to induce bending deformation. A cylindrical rigid body is then placed at the fingertip contact point. Subsequently, the rigid body is displaced downward, and the contact reaction force between the rigid body and the soft robotic finger is controlled. The pressure range for driving the finger’s internal chamber is 0–70 kPa. The relationship between the fingertip force and pressure for both single-chamber and dual-chamber fingers is shown in Figure 1b. As the pressure increases, the fingertip force gradually increases. Before 40 kPa, the fingertip forces of both single-chamber and dual-chamber designs are nearly the same. However, when the driving pressure exceeds 40 kPa, the fingertip force of the dual-chamber driven finger becomes greater than that of the single-chamber driven finger. When the driving pressure reaches 70 kPa, the fingertip force of the dual-chamber driven finger is 2.4 N, while the single-chamber driven finger is 1.22 N. Therefore, the design of the finger with a dual-chamber structure for the distal and proximal ends can enhance the fingertip force.

## 3. Soft Modular Gripper Operational Space Derivation

### 3.1. Modeling of the Soft Finger Bending Deformation

**Figure 2:**
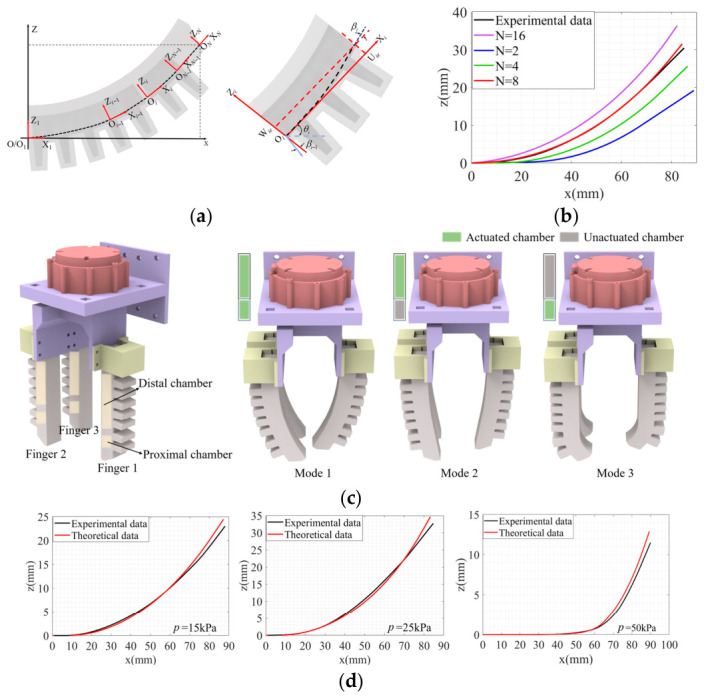
Deformation modeling of soft fingers: (**a**) Modeling coordinate frame of the modular finger. (**b**) Influence of discrete segment number on modular finger bending. (**c**) Three actuation modes of the soft modular gripper: Mode 1—simultaneous proximal and distal actuation; Mode 2—actuation of the proximal section only; Mode 3—actuation of the distal section only. (**d**) Comparison of theoretical and experimental centerline values for three actuation modes under different pressures: Mode 1 *p* = 15 kPa; Mode 2 *p* = 25 kPa; Mode 3 *p* = 50 kPa.

The configuration of the three fingers of the soft modular gripper is shown in Figure 2a. Fingers 2 and 3 are arranged side by side, with a spacing of 4.6 cm between them. Finger 1 is positioned opposite to Fingers 2 and 3, aligned with the centerline of the gap between them, at a distance of 10 cm. To enable grasping control of the soft modular gripper, it is first necessary to determine its operational workspace. The fingers, made of AB silicone, exhibit nonlinear bending deformation under pneumatic actuation. Therefore, a mathematical model of the operational workspace of the soft modular gripper is required. In this paper, the deformation of the finger is modeled by combining the double-layer plate theory with the constant curvature assumption. Based on this, the D-H parameters of the soft modular gripper are established, enabling the determination of its operational workspace. As shown in Figure 2a, establish a coordinate system at the midpoint of the fixed end of the finger at point O, and discretize the finger’s bending deformation into *N* segments. The rotation angle of the *i*-th discrete unit is expressed as follows:

(1) $$\theta_{i}=\sum_{1}^{N}\beta_{i}(i=[2,\ldots ,N]),\theta_{1}=0$$

where $\theta_{i}$ is rotation angle in the fixed coordinate system (O-XYZ), and $\beta_{i}$ is the rotation angle in the local coordinate system (O-X*_i_*Y*_i_*Z*_i_*). The initial bending angle $\theta_{1}$ is assumed to be zero.

Under the assumption of Euler-Bernoulli kinematics (where the normal remain straight and perpendicular to the mid-surface before and after deformation), the plane displacement field of the *i*-th element in three-dimensional space can be expressed as a polynomial function containing undetermined coefficients:

(2) $$\begin{array}{l} u_{i0}=b_{i1}x+b_{i3}x^{3},\;v_{i0}=c_{i1}y,\;w_{i0}=d_{i2}x^{2}\\ \left(x\in [0,L_{i}],y\in [\frac{-C}{2},\frac{C}{2}],i\in [1,\ldots ,N]\right) \end{array}$$

where $u_{i0}$, $v_{i0}$, and $w_{i0}$ represent the displacements in the X, Y, and Z directions in the fixed coordinate system, respectively. And *b_i_*_1_, *b_i_*_3_, *c_i_*_1_, and *d_i_*_2_ are polynomial coefficients.

The displacement $U_{0}(u_{i0},v_{i0},w_{i0})$ can be calculated from the strain as follows:

(3) $$\left\{\begin{array}{l} \;\varepsilon_{ix}=(du_{i0}/dx)+\frac{1}{2}{\left(dw_{i0}/dx\right)}^{2}-z(d^{2}w_{i0}/dx^{2})\\ \;\varepsilon_{iy}=dv_{i0}/dy\\ \gamma_{ixy}=(du_{i0}/dy)+(dv_{i0}/dx)+(dw_{i0}/dx)(dw_{i0}/dy)-z(d^{2}w_{i0}/dydx) \end{array}\right.$$

Based on the theory of small deformation elasticity, the stress–strain relationship of the soft finger is expressed using the generalized Hooke’s law as follows:

(4) $$\left\{\begin{array}{l} \sigma_{1}\\ \sigma_{2}\\ \sigma_{3} \end{array}\right\}=\left[\begin{array}{ccc} Q_{11} & Q_{12} & 0\\ Q_{12} & Q_{22} & 0\\ 0 & 0 & Q_{66} \end{array}\right]\left\{\begin{array}{l} \varepsilon_{1}\\ \varepsilon_{2}\\ \varepsilon_{3} \end{array}\right\}$$

Although the soft finger exhibits large bending deformation macroscopically, the finger is discretized into multiple short segments in the modeling. For each individual segment, the rotation angle and local deformation are sufficiently small, which strictly satisfies the applicable conditions of small-deformation elasticity, Euler-Bernoulli kinematics, and generalized Hooke’s law. Meanwhile, the thickness of the finger is much smaller than its in-plane dimensions, which conforms to the plane-stress assumption in classical laminated plate theory. Therefore, these assumptions are reasonable for the deformation analysis of the soft finger.

Introduce the plane stress assumption from classical laminated plate theory, which neglects the effects of transverse shear stress and normal stress, making the corresponding strain energy component zero. Assume that the driven part of the soft finger is the fluid layer, the undriven part is the constraint layer, and that the constraint layer remains unchanged during the driving process. The strain energy of the constraint layer and fluid layer for the *i*-th unit is expressed as follows:

(5) $$E_{\mathrm{CL}(i)}=\int_{-H_{4}}^{0}\int_{-C/2}^{C/2}\int_{0}^{L_{i}}(\frac{1}{2}{Q_{11}}^{\left(CL\right)}{\varepsilon_{ix}}^{2}+{Q_{12}}^{\left(CL\right)}\varepsilon_{ix}\varepsilon_{iy}+\frac{1}{2}{Q_{22}}^{\left(CL\right)}{\varepsilon_{iy}}^{2})dxdydz$$

(6) $$E_{\mathrm{FL}(i)}=\int_{0}^{H_{2}}\int_{-C_{1}/2}^{C_{1}/2}\int_{0}^{L_{i}}(\frac{1}{2}{Q_{11}}^{\left(FL\right)}{\varepsilon_{ix}}^{2}+{Q_{12}}^{\left(FL\right)}\varepsilon_{ix}\varepsilon_{iy}+\frac{1}{2}{Q_{22}}^{\left(FL\right)}{\varepsilon_{iy}}^{2})dxdydz$$

where *E*_CL(*i*)_ and *E*_FL(*i*)_ represent the strain energy of the constraint layer and the fluid layer, respectively. ***Q*** is the stiffness matrix, which can be calculated using the elastic modulus and Poisson’s ratio.

(7) $$Q_{11}=\frac{E_{1}}{1-v_{12}v_{21}},\;Q_{12}=\frac{v_{12}E_{2}}{1-v_{12}v_{21}},\;Q_{22}=\frac{E_{2}}{1-v_{12}v_{21}}$$

According to the principle of energy conservation, it is assumed that the work done by the pressure is completely converted into the strain energy of the finger’s bending:

(8) $$\frac{\partial \left(\sum_{1}^{N}E_{\left(i\right)}\right)-\partial \left(\sum_{1}^{N}W_{P(i)}\right)}{\partial k_{i}}=0$$

where *E*_(*i*)_ and *W_P_*_(*i*)_ represent the strain energy and the work done by the pressure for the *i*-th segment, respectively. ***k****_i_* is the coefficient in the displacement polynomial *i*-th segment.

The work done by the pressure is calculated as follows:

(9) $$W_{P(i)}=\frac{P_{0}V_{i0}-P_{f}V_{if}}{\gamma -1}$$

where *P*_0_ and *P_f_* represent the initial and final driving pressures, respectively. And *V_i_*_0_ and *V_if_* represent the initial and final driving volumes, respectively. γ is air adiabatic coefficient, which is assumed to be equal to 1.4.

By combining Equations (1)–(9) and using the Rayleigh-Ritz method, the deformation centerline of the soft finger is obtained. As shown in Figure 2b, the centerline of the soft finger’s bending at different segments, with the two chamber driving pressures set to 25 kPa, is compared with the experimentally obtained centerline. The theoretical centerline of the soft finger becomes increasingly curved as the number of segments increases. The theoretical value when the soft finger is discretized into 8 segments is the closest to the experimental result. Therefore, in calculating the soft finger’s behavior under different working modes, it is assumed that each finger is discretized into 8 segments.

As shown in Figure 2c, the soft finger has three working modes: Mode 1 is to drive both chambers of the finger simultaneously with the same pressure; Mode 2 is to drive only the proximal end of the finger; Mode 3 is to drive only the distal end of the finger. For the three working modes, the theoretical and experimental values of the finger’s bending centerline are compared, as shown in Figure 2d. The driving pressures for Modes 1, 2, and 3 are 15 kPa, 25 kPa, and 50 kPa, respectively. To quantitatively evaluate the modeling accuracy, the mean absolute error (MAE) and root mean square error (RMSE) are calculated. For Mode 1, the MAE is 0.41 mm and the RMSE is 0.63 mm; for Mode 2, the MAE is 0.50 mm and the RMSE is 0.78 mm; for Mode 3, the MAE is 0.26 mm and the RMSE is 0.51 mm. Theoretical values and experimental values are generally similar, allowing the derived model to be used for establishing the operational space of the soft finger.

### 3.2. Operational Space of the Gripper

**Figure 3:**
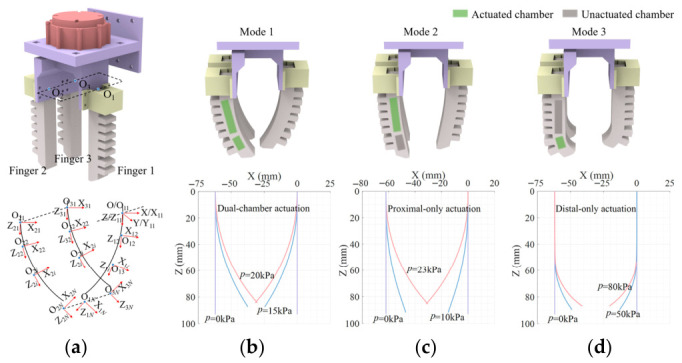
The workspace of the gripper under different modes: (**a**) Coordinate system for the gripper. (**b**) Workspace of the gripper Mode 1. (**c**) Workspace of the gripper Mode 2. (**d**) Workspace of the gripper Mode 3.

The gripper has three working modes. Therefore, a spatial coordinate system for the gripper is established, and using the D-H parameter method, the operational space for different working modes is obtained. As shown in Figure 3a, the spatial coordinate system for the gripper is established. O_1_, O_2_, and O_3_ represent the center points of the fixed ends of the three fingers, respectively. The global coordinate system (O-XYZ) is established at point O_1_, and local coordinate systems ($O_{ij}{\text{-}X}_{ij}Y_{ij}Z_{ij},i\in \left[1,2,3\right],j\in \left[1,2,\ldots ,N\right]$) are established at each discrete point on the fingers. By using the secondary transformation coordinate system, the centerline of the soft finger can be expressed in the fixed coordinate system. The soft finger undergoes planar bending. By first rotating around the Z*_i_*_(*i*−1)_-axis and then translating along the X*_i_*_(*i*−1)_-axis, the coordinates of the soft finger can be transformed into the fixed coordinate system.

The centerline of the gripper in the homogeneous coordinate system is expressed as follows:

(10) $$T_{ij}=\left[\begin{array}{cccc} \cos\beta_{ij} & -\sin\beta_{ij} & 0 & l_{ij}\cos\beta_{ij}\\ \sin\beta_{ij} & \cos\beta_{ij} & 0 & l_{ij}\sin\beta_{ij}\\ 0 & 0 & 1 & 0\\ 0 & 0 & 0 & 1 \end{array}\right]$$

where $\beta_{ij}$ and *l_ij_* represent the rotation angles and translation distances for transforming the coordinates of the *i*-th finger from the (*j* − 1)-th joint coordinate system to the *j*-th joint coordinate system.

The operational spaces of the gripper under the three working modes are illustrated in Figure 3b–d. Through theoretical derivation, the maximum driving pressures during the gripping phase for modes 1, 2, and 3 were determined to be 20, 23, and 80 kPa, respectively. These maximum driving pressures refer to the pressures when the fingertips reach a specific position. At these pressure levels, the fingertips of the three fingers align in a straight line. If the pressure exceeds the corresponding maximum value, the gripper will lose its ability to grasp objects effectively.

## 4. Target Recognition and Localization Based on the Improved YOLOv8

### 4.1. YOLOv8 Improvements and Manual Dataset Annotation

**Figure 4:**
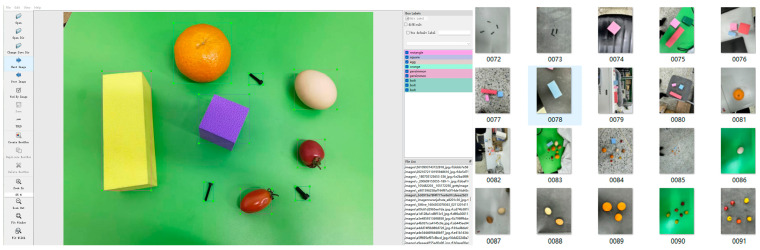
Manually created dataset and labeled annotations.

In this paper, the gripper is primarily used to grasp five types of objects: bolts, cherry tomatoes, oranges, eggs, and toy building blocks. As shown in Figure 4, a total of 7406 images were collected and manually annotated with LabelImg to build a custom dataset for the above five objects of different sizes. The dataset was divided into training and testing sets at a ratio of 7:3. The YOLOv8n model was selected and trained for 200 epochs with a batch size of 16, a momentum of 0.937, an initial learning rate of 0.01, and a weight decay of 0.0005. The hardware environment was equipped with an NVIDIA GeForce RTX 3050 GPU and an Intel Core i7-12700 CPU, along with the software configuration of Python 3.9, CUDA 11.3 and PyTorch 1.12.0.

**Figure 5:**
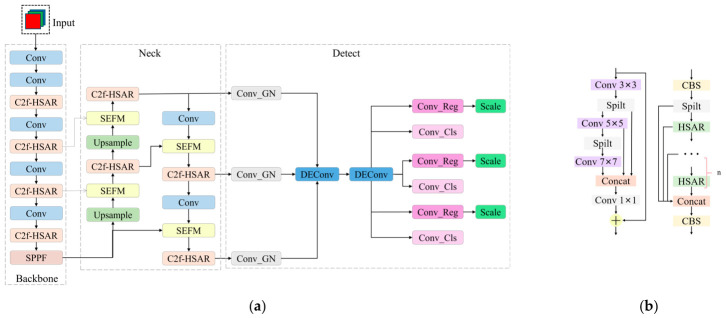

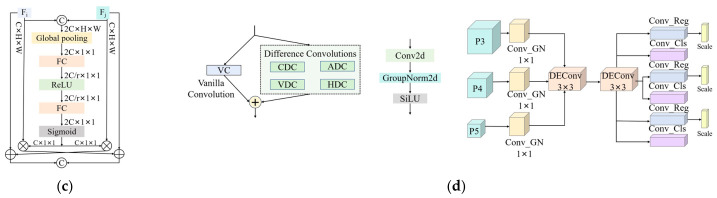
Improvements in YOLOv8: (**a**) Architecture of the improved YOLOv8. (**b**) Improvements in the C2f-HSAR and HSAR modules. (**c**) Improvements in the SEFM. (**d**) Improvements in the SCDH module.

The C2f, SEFM, and SCDH modules were enhanced based on the YOLOv8 architecture, as illustrated in Figure 5. Figure 5a shows the structure of the improved YOLOv8. The C2f module constructs the Bottleneck module by stacking 3 × 3 standard convolutions. However, the use of a single-scale convolution kernel limits the network’s receptive field, making it difficult to effectively capture multi-scale object features. To address this issue, we propose the Hierarchical Split-Aggregate Residual (HSAR) module and construct the C2f-HSAR network architecture, as illustrated in Figure 5b. In the C2f-HSAR network, the HSAR module replaces the original Bottleneck module. The HSAR module first performs feature extraction using a 3 × 3 standard convolution, and then evenly splits the output features along the channel dimension. The first half of the features pass sequentially through 5 × 5 and 7 × 7 convolutions, thereby expanding the receptive field, while the second half of the features, processed by the 3 × 3 convolution, retain high-resolution detail. By concatenating the global semantic features from the 7 × 7 convolution, the local contextual features from the 5 × 5 convolution, and the detail features preserved by the 3 × 3 convolution, cross-scale fusion is achieved. After restructuring the features with a 1 × 1 convolution, the output is added to the input residual, enhancing the network’s multi-scale perception and feature fusion capabilities.

In feature pyramid networks, multi-scale feature fusion is typically achieved using the Channel Concatenation (Concat) module, which directly concatenates feature maps from different layers along the channel dimension. However, the Concat module assigns equal weights to all input feature channels, failing to account for the differences between shallow detail features and deep semantic features. This limitation may result in insufficient information fusion, leading to issues such as detail loss, false positives, or missed detections. To address this, we propose the Squeeze-and-Excitation Fusion Module (SEFM), whose core idea is to dynamically learn channel weights through the SE attention mechanism. This enhances important channels and suppresses redundant ones, optimizing the feature fusion process, as shown in Figure 5c. In the implementation of the SEFM, the input features are first aligned along the channel dimension, followed by concatenation with the input to the SE module. The SE module begins by performing global average pooling to compress the spatial dimensions of the feature map and then utilizes two fully connected layers to learn the dependencies between channels. The output is a channel weight tensor, which is split into two parts. These parts are then multiplied by the original input features to generate the weighted features. Finally, by adding the weighted shallow features to the deep features and the weighted deep features to the shallow features, bidirectional feature compensation is achieved.

Although the decoupled detection head used in YOLOv8 improves detection performance by independently handling classification and regression tasks, its multi-layer convolutional structure enhances feature representation but also introduces issues such as parameter redundancy and a lack of multi-scale feature interaction. To address these challenges, we propose the Shared Convolutional Detection Head (SCDH) module, which optimizes the performance of the detection head by sharing convolutional parameters and enhancing feature interactions. The core idea of the SCDH module is to fuse multi-scale feature maps from the neck network via the parameter-shared Detail Enhanced Convolution (DEConv) [23] and introduce Group Normalization (GN) to improve task performance. As shown in Figure 5d, the SCDH module first uses a 1 × 1 convolution combined with GN to unify the feature channel dimensions, reducing computational complexity while eliminating feature distribution discrepancies between layers. It then aggregates spatial context information through a DEConv cascading structure with shared weight parameters. The DEConv consists of five parallel convolutions: standard convolution, center difference convolution (CDC), horizontal difference convolution (HDC), vertical difference convolution (VDC), and angular difference convolution (ADC). DEConv integrates these five branches to capture global intensity features and multi-directional gradient details. Specifically, the standard convolution extracts global intensity features; CDC extracts central edge details; HDC and VDC capture horizontal and vertical gradient features; ADC extracts gradient features along 45° and 135° diagonal directions to supplement edge information. The outputs of the five parallel branches are fused by element-wise summation to generate the final output feature map of DEConv. The feature fusion enhances the response to edge features.

### 4.2. Ablation Study

To evaluate improvements to YOLOv8, six ablation experiments were conducted to assess the impact of the C2f-HSAR, SEFM, and SCDH modules. Metrics included mAP, Params, and GFLOPS. As shown in Table 3, C2f-HSAR improved mAP by 1.5%, highlighting the benefit of multi-scale convolutions. SEFM further increased mAP by 2.1% via the SE attention mechanism, with a slight rise in parameters.

The SCDH module slightly improved accuracy, confirming DEConv’s effectiveness in detail extraction. Combining C2f-HSAR and SEFM boosted all metrics, showing synergy. The final improved model increases mAP by 3.4%, reduces parameters by 29.9%, and decreases computational cost by 28.4%, demonstrating that multi-scale processing, attention-based feature reorganization, and shared detection heads enhance detection while lowering complexity.

After 200 epochs of iterative training, the improvement effect of the model can be clearly observed. The precision, recall, and F1-score of the original model are 87.5%, 87.4%, and 87.4%, respectively. The improved model achieves a precision of 89.0%, a recall of 89.2%, and an F1-score of 89.1%. Compared with the original model, the improved algorithm delivers enhanced performance.

**Figure 6:**
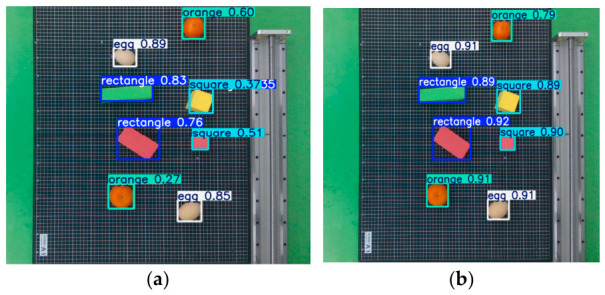
Comparison of detection results before and after optimization: (**a**) Original YOLOv8. (**b**) Improved YOLOv8.

As shown in Figure 6, the original YOLOv8 model exhibits false detection when recognizing multiple objects, mistakenly identifying an egg as an orange. In contrast, the improved model eliminates such errors and achieves a higher overall recognition accuracy, demonstrating the effectiveness of the proposed enhancements.

## 5. Experiments

### 5.1. Construction of the Grasping Platform

**Figure 7:**
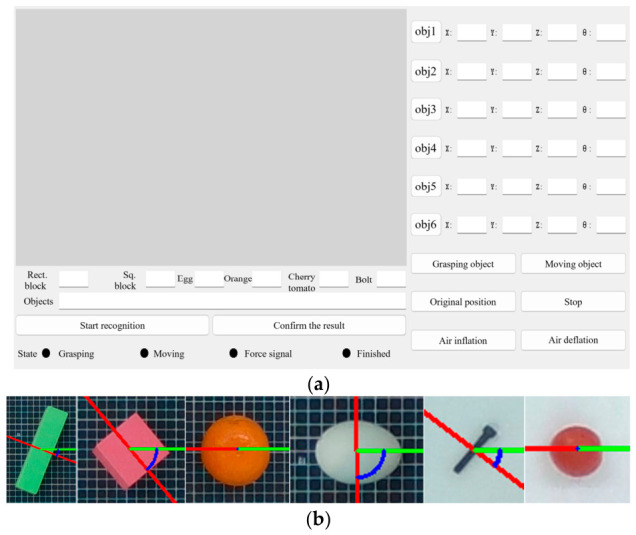
Integrated grasping platform design: (**a**) Grasping platform. (**b**) Object pose estimation. Red line: short axis of bounding rectangle; green line: gripper’s initial opening; blue arc: angle between them.

The control platform of the grasping system integrates a vision sensor, pressure sensor, gripper actuator, object detection algorithm, driving unit, and mobility method. A PyQT5-based interactive interface is developed for visualizing and controlling the gripper, divided into four sections: image display, object recognition and localization, hand movement, and operation, as shown in Figure 7a.

The input image is first preprocessed by converting it from color to single-channel grayscale. Otsu’s algorithm is then applied for binarization to enhance the object contour. The areas of all contours are calculated, and the largest one is selected. By extracting the edge features of this contour, the minimum bounding rectangle is fitted, which is then used to determine the object’s pose. As shown in Figure 7b, the image is converted to grayscale and binarized using Otsu’s algorithm to enhance object contours, followed by Gaussian filtering for edge feature processing. The shorter axis of the bounding rectangle (red) is used as the object orientation reference, and the angle between it and the gripper’s initial opening (green) is calculated (blue arc), enabling pose estimation for guiding the grasping action of the soft modular gripper.

The D435i camera captures RGB-D data at 1280 × 720 resolution, with intrinsic parameters calibrated using Zhang’s calibration method in MATLAB R2023a. Extrinsic parameters are determined via the EPNP algorithm, mapping the camera coordinate system to the gripper’s. The improved YOLOv8 model detects objects, providing key information (category, bounding box, center coordinates, and pose) displayed on the interface for grasping.

The camera intrinsic matrix M is as follows:

(11) $$\left[\begin{array}{ccc} 909.6575 & 0 & 653.4960\\ 0 & 909.9615 & 366.0403\\ 0 & 0 & 1 \end{array}\right]$$

### 5.2. Grasping Experiments

**Figure 8:**
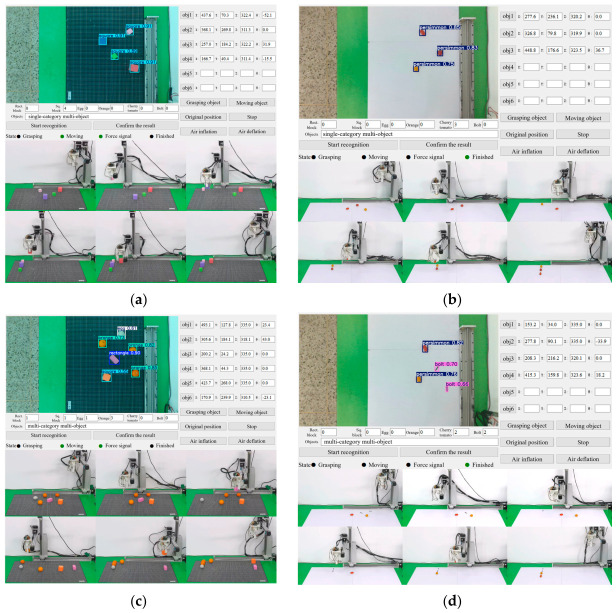
Grasping object experiments: (**a**) Two different-sized square building blocks. (**b**) Cherry tomatoes. (**c**) Mixed grasping experiment with eggs, building blocks, and oranges. (**d**) Mixed grasping experiment with cherry tomatoes and bolts.

To comprehensively evaluate the accuracy of object recognition and grasping, experiments were conducted using five types of target objects-square building blocks, oranges, eggs, cherry tomatoes, and bolts. The masses and dimensions of the five test objects are as follows. Building blocks have side lengths of 35 mm and 50 mm with a mass of approximately 10 g. Oranges have a diameter of about 35 mm with a mass of approximately 50 g. Eggs have a length of about 43 mm with a mass of approximately 60 g. Cherry tomatoes have a diameter of about 25 mm with a mass of approximately 15 g. Bolts have a diameter of 6 mm, a length of 30 mm, and a mass of approximately 6 g. These included single-category multi-object grasping and multi-category multi-object grasping. The process of the grasping experiments is illustrated in Figure 8.

Figure 8c,d illustrate two types of mixed-object grasping experiments. One mixed-object grasping experiment was conducted involving egg, rectangular building block, rectangular building block, and oranges. The building blocks were grasped using Mode 3, whereas the oranges and eggs were first grasped with Mode 1, and then switched to Mode 3 for gripping. The other experiment was a mixed-object grasping experiment involving cherry tomatoes and bolts. The soft modular gripper initially used Mode 3 for grasping, followed by a switch to Mode 2 to grasp the cherry tomatoes and bolts. Through experiments on the soft modular gripper’s recognition and grasping of different objects, the accuracy of the recognition and the controllability of the grasping were demonstrated.

For lightweight objects such as square blocks and cherry tomatoes, a pinching grasp strategy (Mode 3) was adopted, as shown in Figure 8a. The system is capable of recognizing and localizing four square building blocks-two with a side length of 35 mm and two with a side length of 50 mm. For these lightweight blocks, a pinching grasp strategy with distal actuation was employed. The distal driving pressure was set to 80 kPa for the 35 mm blocks and 50 kPa for the 50 mm blocks. The grasping of cherry tomatoes was performed using a sequential actuation strategy, where the distal end (Mode 3) was actuated first to establish initial contact, followed by proximal actuation (Mode 2) to secure the grasp. As shown in Figure 8b, the distal end was first actuated to its maximum pressure of 80 kPa to approach the edge of the cherry tomato. Then, the proximal end was actuated with a pressure of 10 kPa to gently pinch and secure the tomato, after which the object was lifted and moved.

### 5.3. Repetitive Grasping Experiments

**Figure 9:**
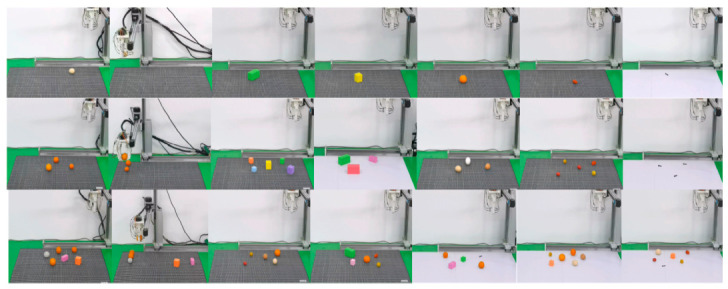
Repetitive grasping experiments design.

Repetitive grasping experiments were conducted for the five target object categories, including single-category single-object grasping, single-category multi-object grasping, and multi-category multi-object grasping, as shown in Figure 9. The number of objects in each multi-object task ranged from 3 to 6, and 50 repeated trials were performed for each category. The experimental results are summarized in Table 4, Table 5, Table 6 and Table 7.

Table 4 presents the results of single-category single-object grasping. One object was grasped per trial, with 10 repeated trials for each object category, totaling 50 trials across five object types. The grasping task succeeded 47 times, achieving an overall success rate of 94%. A total of three failures occurred in single-object grasping: one failure for cherry tomatoes and two failures for bolts.

Table 5 summarizes the results of single-category multi-object grasping. Ten trials were conducted for each object category, with 5 square blocks, 6 cherry tomatoes, 4 eggs, 6 oranges, and 3 bolts per trial, respectively. A total of 240 grasps were performed, among which 233 were successful, yielding a success rate of 97.1%. A total of seven failures were observed: two for cherry tomatoes and five for bolts.

Table 6 shows the results of multi-category multi-object grasping. The five types of objects were randomly mixed, with 4–6 objects per scene. Five typical grasping scenes were designed, each repeated 10 times. Scene 1 contained one egg, one orange, one cherry tomato, and one block. Scene 2 contained one of each object including a bolt. Scene 3 contained two eggs, two oranges, and two blocks. Scene 4 contained two oranges, two cherry tomatoes, and one block. Scene 5 contained two cherry tomatoes, two blocks, and two bolts. A total of 260 grasps were completed, with 250 successful attempts, corresponding to a success rate of 96.2%. Failures included one for blocks, three for cherry tomatoes, and six for bolts.

Table 7 summarizes the overall statistics of all repetitive grasping experiments. Among the total 550 grasping trials across all five categories, 530 were successful, resulting in an overall success rate of 96.4%. Most failures occurred during bolt grasping, mainly because the small diameter of bolts led to uneven contact forces among the three fingers under the same driving pressure, leading to grasping instability or dropping during movement. A small number of failures occurred for cherry tomatoes due to their smooth surfaces, which caused slippage even after successful initial grasping.

## 6. Conclusions and Future Work

This paper presents a pneumatic modular soft gripper based on visual object detection and constructs an integrated control platform for the grasping system to achieve object recognition, localization, and grasping. A distal-proximal dual-chamber structure is proposed for the modular soft finger, enabling the soft gripper to possess three grasping modes. Based on the classical laminated plate theory, the relationship between the curvature centerline of the finger and the driving pressure is established. The D-H parameter method is adopted to establish the spatial coordinate system of the soft gripper, and the workspace of the gripper under the three modes is derived. A dataset is constructed for five types of objects, namely oranges, eggs, building blocks, bolts, and cherry tomatoes. The C2F-HSAR, SEFM, and SCDH modules in YOLOv8 are improved, and ablation experiments demonstrate that the improved algorithm increases mAP by 3.4%, reduces Params by 29.9%, and decreases GFLOPS by 28.4%. An integrated control platform combining vision, soft gripper control, and state detection is built. Grasping experiments are carried out on single-category objects, multi-category objects, and repetitive grasping. The experimental results show that the grasping success rate of the soft gripper reaches 96.4%, which validates the effectiveness of the improved recognition algorithm and the controllability of the proposed modular soft gripper.

The soft modular gripper is made of silicone, which inherently limits the weight of objects it can grasp, especially when grasping heavier payloads. In repeated grasping experiments, failures frequently occur when grasping bolts, mainly because the same driving pressure leads to uneven force distribution at the tip of each soft finger, resulting in unsuccessful grasping or objects dropping during movement. To address this, a chamber can be integrated into the base of the soft finger and filled with coffee grounds. By applying negative pressure to actuate this chamber, the stiffness of the gripper can be increased, thereby improving its capability to grasp heavier objects. Meanwhile, the fingertip can be extended to mimic the rigid human fingernail structure, and fingerprint-like textures can be added to the finger pad to increase the friction coefficient, thus further enhancing grasp stability. However, although this approach can enhance the gripping force of the soft gripper, it also introduces new theoretical challenges, such as understanding the relationship between the negative pressure applied to the coffee ground chamber and the resulting stiffness of the soft gripper. In addition, the structural modification of the fingertip and finger pad also introduces the nonlinear modeling problem of composite-hardness soft fingers, which needs to be further derived. Therefore, in future research, we will continue to conduct in-depth studies focusing on the soft finger structure and the improvement of gripping force.

## Figures and Tables

**Table 1:** Comparison of soft grippers.

References	Structure Design	Actuation	Grasping Modes	Theoretical Modeling	Perception
Zhang et al. (2023) [4]	Modular integrated Pneu-net structure	Pneumatic	Multi-mode	None	None
Huang et al. (2023) [6]	Configurable modular composite finger	Pneumatic	Multi-mode	Theoretical modeling of grasping force	None
Zhao et al. (2024) [13]	Modular variable stiffness structure	Pneumatic	Dual-mode	Bending mathematical model; Variable stiffness mathematical model	None
Jain et al. (2023) [15]	Reconfigurable workspace modular gripper	Pneumatic	Multi-mode	None	None
Cheng et al. (2022) [17]	Reconfigurable biomimetic pneumatic gripper	Pneumatic	Dual-mode	Bending deformation modeling	Shape and size recognition for fruit objects
Zhang et al. (2024) [19]	Soft gripper with optical tactile palm	Pneumatic	Single-mode	None	Optical tactile sensing
Zhang et al. (2025) [21]	Tactile-finger coordinated soft robotic hand	Pneumatic	Dual-mode	None	Tactile feedback and sensing
This work	Dual-chamber modular finger	Pneumatic	Multi-mode	Bending deformation modeling; D-H workspace modeling	Improved YOLOv8 for object detection and localization

**Table 2:** Structural and material parameters of the soft modular gripper.

Parameter	Value	Parameter	Value	Parameter	Value
*L*	93 mm	*L*_1_	52 mm	*L*_2_	16 mm
*C*/2	20 mm	*H*	24 mm	*H*_1_	12 mm
*H*_2_	12 mm	*H*_3_	5 mm	*H*_4_	2 mm
*t*_1_	2 mm	*t*_2_	3 mm	δ	60°
*E*_1C_	1200 MPa	*E*_2C_	1200 MPa	*E*_1__F_	1.2 MPa
*E*_2F_	1.2 MPa	$\nu_{12}$	0.48	$\nu_{21}$	0.48

Note: Structural parameters are in millimeters (mm), and material parameters follow SI units. *E*_1C_, *E*_2C_ represent the Young’s moduli of the constraining layer; *E*_1F_, *E*_2F_ represent the Young’s moduli of the fluidic layer; $\nu_{12}$ and $\nu_{21}$ are Poisson’s ratios.

**Table 3:** Ablation experiment.

Module	C2f-HSAR	SEFM	SCDH	mAP/%	Params/M	GFLOPS
a	×	×	×	88.7	3.01	8.1
b	√	×	×	90.2	2.60	7.1
c	×	√	×	90.8	3.16	8.3
d	×	×	√	89.1	2.36	6.5
e	√	√	×	91.9	2.76	7.3
f	√	√	√	92.1	2.11	5.8

Note: ‘√’ indicates the inclusion of the module, while ‘×’ indicates its exclusion.

**Table 4:** Single-category single-object grasping results.

Category	Successful/Total Trials	Success Rate (%)
Single-category single-object	47/50	94
Building block	10/10	100
Cherry tomato	9/10	90
Egg	10/10	100
Orange	10/10	100
Bolt	8/10	80

**Table 5:** Single-category multiple-objects grasping results.

Category	Successful/Total Trials	Success Rate (%)
Single-category multi-object	233/240	97.1
Building block (5 pieces)	50/50	100
Cherry tomato (6 pieces)	58/60	96.7
Egg (4 pieces)	40/40	100
Orange (6 pieces)	60/60	100
Bolt (3 pieces)	25/30	83.3

**Table 6:** Multiple-category multiple-objects grasping results.

Category	Successful/Total Trials	Success Rate (%)
Multi-category multi-object	250/260	96.2
Building block	69/70	98.6
Cherry tomato	57/60	95
Egg	40/40	100
Orange	60/60	100
Bolt	24/30	80

**Table 7:** Results of repeatability grasping experiments.

Category	Successful/Total Trials	Success Rate (%)
Single-category single-object	47/50	94
Single-category multi-object	233/240	97.1
Multi-category multi-object	250/260	96.2
Total	530/550	96.4

## Data Availability

Data is contained within the article.
